# Biocompatibility Evaluation of Ampicillin-Loaded Whitlockite for Bone Regeneration

**DOI:** 10.7759/cureus.61461

**Published:** 2024-05-31

**Authors:** Jabeen Nazurudeen, Sinduja Palati, Saravanan Sekaran, Dhanraj Ganapathy

**Affiliations:** 1 Pathology, Saveetha Dental College and Hospitals, Saveetha Institute of Medical and Technical Sciences , Saveetha University, Chennai, IND; 2 Oral and Maxillofacial Pathology, Saveetha Dental College and Hospitals, Saveetha Institute of Medical and Technical Sciences , Saveetha University, Chennai, IND; 3 Prosthodontics, Saveetha Dental College and Hospitals, Saveetha Institute of Medical and Technical Sciences , Saveetha University, Chennai, IND; 4 Prosthodontics, Saveetha Dental College and Hospitals, Saveetha Institute of Medical and Technical Sciences, Saveetha University, Chennai, IND

**Keywords:** whitlockite, hydroxyapatite, regeneration of bone, biocompatibility, antibiotics

## Abstract

Introduction

Whitlockite (WH), a rare phosphate mineral within the apatite group, shows potential for bone regeneration owing to its superior composition and biocompatibility compared to hydroxyapatite. It can serve as a carrier for bioactive molecules, gradually releasing them to stimulate bone growth and expedite healing. This study aims to assess the biocompatibility of antibiotic-loaded WH, focusing on ampicillin, for bone regeneration applications.

Methodology

WH particles loaded with varying concentrations of ampicillin (10 and 25 mM) underwent biocompatibility assessments using the MTT assay. One gram of particles was incubated in 10 mL of culture medium for 24 and 48 hours. Experimental groups included control, WH, WH with ampicillin at 10 mM (WH+A10), WH with ampicillin at 25 mM (WH+A25), and positive control treated with 0.1% Triton X detergent. Subsequently, after a three-day culture period, RunX2 gene expression, indicative of osteoblastic differentiation, was quantified using real-time PCR analysis.

Results

Our research evaluated the bioactivity of WH particles treated with human osteoblastic cells using the MTT assay. While 10 mM ampicillin-loaded WH showed no significant difference in metabolic activity at both 24 and 48 hours, 25 mM ampicillin-loaded WH exhibited a slight reduction in metabolic activity at 24 hours, which normalized by 48 hours. Additionally, we assessed osteogenic potential and showed a significant increase in RunX2 expression with ampicillin-loaded WH, indicating sustained osteogenic properties.

Conclusions

Our study underscores the promising biocompatibility of WH particles by retaining their osteogenic properties even when, loaded with ampicillin, offering a potential avenue for future bone regeneration strategies.

## Introduction

Bone regeneration is a highly intricate physiological process involved in normal fracture healing and continuous remodeling throughout adulthood. In clinical contexts such as trauma, infection, tumor removal, skeletal abnormalities, or compromised regenerative processes like avascular necrosis, atrophic non-unions, and osteoporosis, extensive bone regeneration becomes imperative. Various methods, including autologous bone grafts, growth factors, and tissue engineering, enhance impaired bone regeneration [1]. Current research focuses on refining local and systemic interventions. These efforts aim to overcome the limitations of existing methods, create bone graft substitutes with biomechanical properties resembling natural bone, expedite the overall regeneration process, and address systemic conditions like skeletal disorders and osteoporosis [2].

In dentistry, bone regeneration is critical for procedures such as dental implants, periodontal disease treatment, and ridge preservation. Dental implants require sufficient bone volume for stability, often achieved through bone augmentation techniques. Periodontal disease can lead to bone loss around teeth, necessitating bone regeneration for tissue attachment [3]. Ridge preservation minimizes bone loss post-extraction, facilitating future implant placement. Sinus lift surgery augments bone height in the posterior maxilla, enabling implant placement. Jaw defects require reconstruction through bone grafting or guided bone regeneration, restoring function and aesthetics. By promoting new bone formation and stability, these techniques enable better oral function, aesthetics, and overall patient well-being [4,5].

Hydroxyapatite (HAP), a key component of bone, shares both structure and chemical composition similarities with natural human bone. It is predominantly present in bones and teeth, serving as a vital inorganic component. Due to its exceptional biocompatibility and biodegradability, it has been extensively studied for its potential in bone tissue engineering. HAP is widely utilized in bone tissue remodeling (osteogenesis) as well as in drug delivery systems, where it acts as a drug transporter [6]. Whitlockite (WH), the second most prevalent mineral in living bone, is created through the partial substitution of calcium ions with magnesium ions (about 20% in terms of Mg2+ quantity) within calcium orthophosphate crystals [7]. It shows promise due to its unique composition and properties. Studies demonstrate WH's potential to enhance bone regeneration, particularly in calvarial defects [8]. WH's role, along with HAP, in bone physiology remains under investigation, emphasizing their distinct contributions to bone regeneration. These scaffolds provide a supportive structure for bone cells to adhere, proliferate, and differentiate, aiding in the regeneration of damaged or lost bone tissue. Additionally, researchers are exploring ways to incorporate WH into innovative tissue engineering approaches and gene therapies. By understanding the biological significance and potential applications of WH in bone regeneration, scientists aim to develop advanced strategies that can effectively address the challenges associated with impaired bone healing and various skeletal disorders [9,10].

Ampicillin, an antibiotic, has potential applications in bone regeneration and tissue engineering due to its antibacterial properties. Some studies have investigated the use of ampicillin in combination with other materials for its role in promoting bone growth and regeneration. The idea is that ampicillin, in addition to its antibacterial properties, might have some positive effects on bone cells or the bone regeneration process [11]. One possible avenue of exploration is the use of antibiotic-loaded biomaterials or scaffolds in orthopedic applications. These biomaterials could release ampicillin locally, providing both an antimicrobial effect and potentially aiding in bone regeneration [12]. The controlled release of antibiotics within the bone defect site may help prevent infections while supporting the healing process. This study evaluates the biocompatibility of antibiotic-loaded WH for bone regeneration, contributing to the advancement of orthopedic applications.

## Materials and methods

Preparation of WH

The synthesis of WH was conducted utilizing an in-house methodology, referencing Amirthalingam et al., employing an aqueous solution maintained below the boiling temperature of water. Calcium hydroxide (Ca(OH)_2_) and magnesium hydroxide (Mg(OH)_2_) were initially mixed in distilled water (500 mL) at 80 °C [13]. While vigorously stirring, an aqueous solution of phosphoric acid (H_3_PO_4_) was gradually added to the mixture using a distal burette at a rate of 12.5 mL/minute. After aging for 24 hours, the precipitates were collected using a filter press and subsequently freeze-dried.

Cell culture

Human osteoblastic cells (MG63) were obtained from the National Center for Cell Sciences (NCCS), Pune, India. The cells were maintained under normal standard culture conditions at 5% CO_2_ and 10% fetal bovine serum (FBS)-containing medium. Cells were cultured, trypsinized, passaged, and used for further studies. The cells were checked for morphological analysis to confirm the cell viability.

MTT assay

WH particles (1 g) were soaked in Dulbecco's Modified Eagle Medium (DMEM) containing 10% FBS for 24 hours to obtain a conditioned medium. Similarly, ampicillin-loaded WH particles at concentrations of 10 and 25 mM underwent the same procedure. Human osteoblastic cells were treated with 1 mL of conditioned medium for 24 and 48 hours. Triton X-100-treated cells served as positive controls, while untreated cells served as controls. After incubation, the medium was removed and 0.5% MTT solution was added and incubated for three to four hours at 37 °C. After the incubation period, the MTT solution was discarded and 100 microL of dimethyl sulfoxide (DMSO) was added and incubated at dark conditions for one hour. The absorbance was measured at 590 nm after solubilization with DMSO.

Real-time PCR

**Figure 1:**
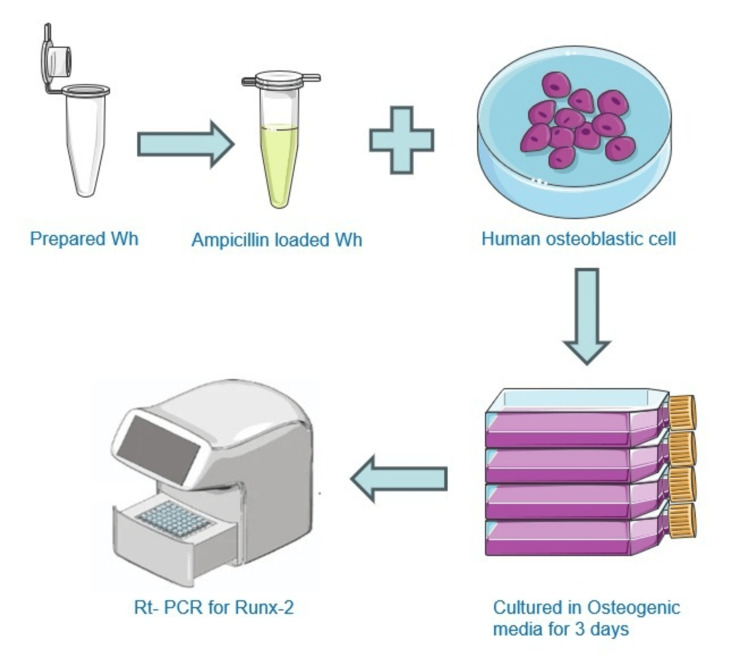
Schematic representation of the method followed. The picture contains altered images from Servier Medical Art, licensed by a Creative Commons Attribution 3.0 Unported License. RT-PCR, Reverse Transcription Polymerase Chain Reaction

Human osteoblastic cells were cultured in an osteogenic induction medium for three days, and total RNA was extracted using the TRIzol method. Purified RNA was reverse-transcribed into cDNA, and Reverse Transcription Quantitative Polymerase Chain Reaction (RT-qPCR) was performed using SYBR green and specific primers for target genes (Runx2). Relative mRNA expression levels were analyzed using the 2−ΔΔCq method. The schematic representation of the methodology is depicted in Figure 1.

Statistical analysis

Experiments were conducted in triplicate (*n *= 3). Statistical significance was determined using a paired t-test in SPSS software, with a *P*-value ≤ 0.05 considered significant. Results were presented as mean ± standard deviation. An asterisk (*) denotes a significant increase compared to control, while # denotes a significant decrease. A *P*-value equal to or less than 0.05 was deemed indicative of statistical significance.

## Results

**Figure 2:**
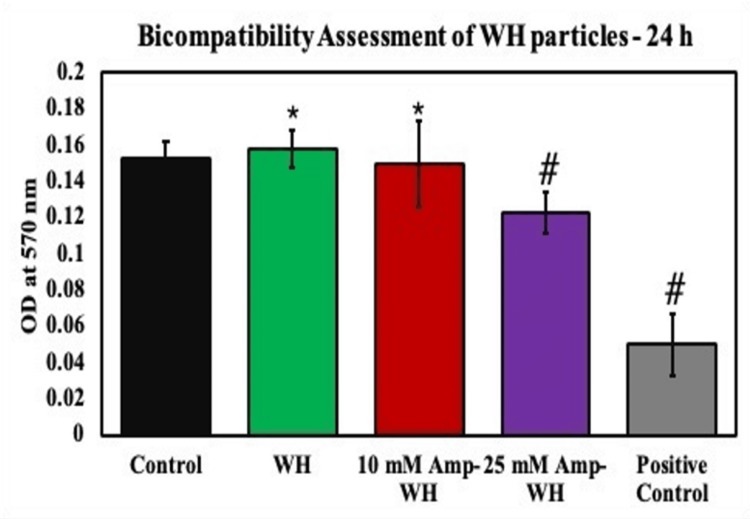
Biocompatibility assessments of cells exposed to ampicillin-loaded whitlockite (Amp-WH) for 24 hours by MTT assay. Treatment groups were compared with untreated control. The graph shows significant changes in the metabolic activity compared to the control. ^*^Indicates a significant increase compared to control. ^#^Indicates a significant decrease compared to control.

The cytotoxicity of the composites was evaluated using the MTT assay, which measures the metabolic activity of cells. None of the tested composites were found to induce damage to the cell membrane, as depicted in Figure 2, which illustrates the 24-hour MTT assay. A noticeable alteration in metabolic activity compared to the control was observed, indicating significant changes in cell viability.

**Figure 3:**
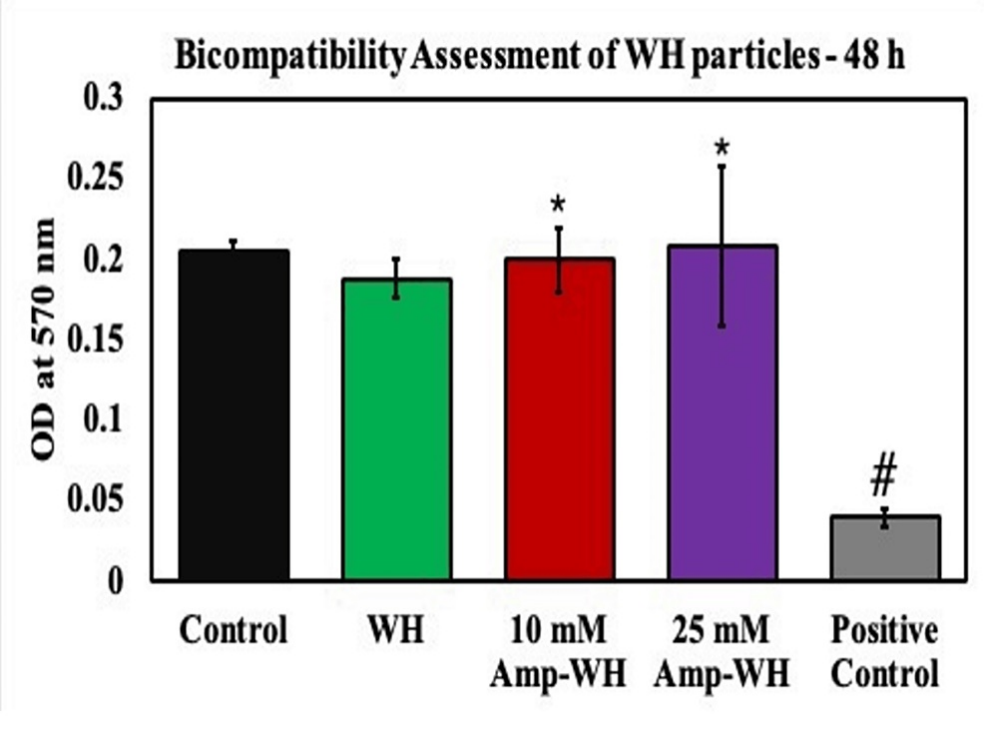
Biocompatibility assessments of cells exposed to ampicillin-loaded whitlockite (Amp-WH) for 48 hours by MTT assay. Treatment groups were compared with untreated control. There was no alteration in the metabolic activity of cells in all the groups indicating the cytofriendly nature. *Indicates a significant increase compared to control. ^#^Indicates a significant decrease compared to control.

At the 48-hour mark (Figure 3), no significant toxicity toward osteoblastic cells was noted, suggesting the inert nature of the particles over this time frame.

**Figure 4:**
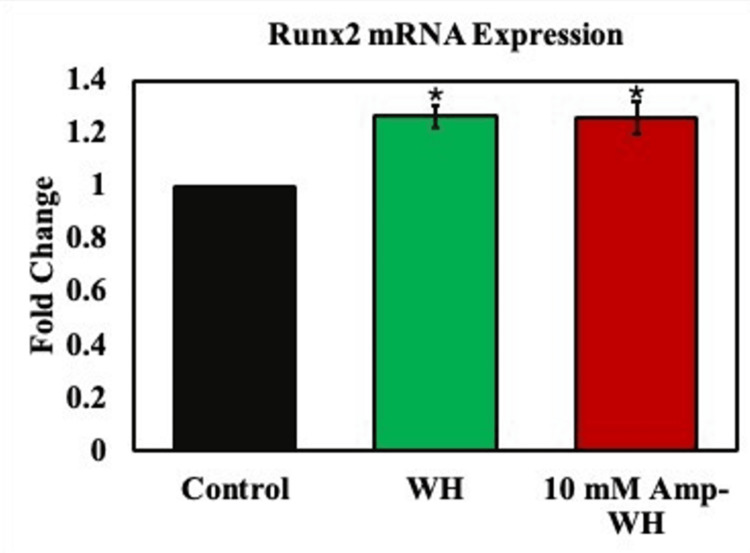
RunX2 mRNA expression analysis by qPCR in MG-63 cells. The treatment group ampicillin-loaded whitlockite (Amp-WH) were compared with untreated control. *Indicates a significant increase compared to control. qPCR, Quantitative Polymerase Chain Reaction

To further investigate the osteogenic potential of the particles, human osteoblastic cells were treated with a conditioned medium obtained from the particles for three days. Subsequently, the expression of the RunX2 gene was analyzed using real-time RT-PCR, as shown in Figure 4. The results indicate a substantial increase in RunX2 expression upon treatment with WH particles, with a similar effect observed in the groups treated with ampicillin-loaded WH particles. This suggests that the addition of ampicillin did not compromise the osteogenic properties of WH particles.

In the context of potential applications in drug delivery, medical treatments, and bone tissue engineering, the incorporation of ampicillin into WH warrants a thorough assessment of its biocompatibility. Our findings indicate that the combination of WH and ampicillin exhibits no cytotoxic effects, with metabolic activity levels at 24 hours (0.1500 ± 0.0236) and 48 hours (0.1993 ± 0.0206) comparable to those of control samples.

This non-toxicity profile, coupled with demonstrated osteogenic properties, underscores the potential utility of WH-ampicillin composites for bone regenerative purposes. Further studies are needed to validate these findings and explore their translational potential in clinical settings.

## Discussion

The biocompatibility of antibiotic ampicillin-loaded WH designed for bone regeneration was thoroughly assessed in this study. Our MTT assay results point towards the noncytotoxic nature of the composites, corroborating with similar findings in the literature that advocate the biocompatible characteristics of WH in bone tissue engineering applications [14,15]. The preserved cell membrane integrity and sustained metabolic activity in osteoblastic cells at both 24-hour and 48-hour intervals confirm the safe interaction of the composite with the cellular environment. Particularly, the unaltered metabolic activity in osteoblastic cells treated with the antibiotic-loaded WH composites at 24-hour and 48-hour time points aligns with findings by other researchers who have previously noted the cytocompatibility of implant materials containing antimicrobial agents [16]. This is crucial for the application of WH in a clinical setting since it suggests that the composite maintains cellular viability over the critical initial period post-implantation.

The amalgamation of WH and ampicillin presents a promising avenue for the development of a controlled-release drug delivery system. Leveraging WH's porous structure, there exists the potential for sustained release of ampicillin, thereby facilitating prolonged therapeutic effects [1]. This feature suggests its applicability in localized antibiotic delivery, particularly advantageous in scenarios necessitating targeted antibiotic therapy, such as the management of localized infections. In orthopedics, a WH-based material laden with ampicillin could serve a dual purpose, functioning as a bone graft material to promote bone regeneration while concurrently offering antibacterial properties to prevent or treat infections at the implantation site.

Our study findings are in alignment with previous research endeavors that have explored the utility of bioactive materials in bone repair [1]. Notably, hydroxyapatite and WH have garnered attention due to their presence in hard tissues like bones and teeth. In a related study, hollow microspheres of hydroxyapatite and WH were synthesized via a microwave-assisted hydrothermal method and assessed for in vitro biocompatibility. The superiority of the WH composite membrane in promoting the proliferation and osteogenic differentiation of human mesenchymal stem cells compared to hydroxyapatite/chitosan was evident. Additionally, it exhibited substantial improvement in bone regeneration in calvarial defects, underscoring its potential in tissue engineering, particularly in calvarial repair [1].

Furthermore, the elevation in RunX2 gene expression implied promising osteogenic abilities of the antibiotic-loaded WH composites. RunX2, being a pivotal transcription factor for bone differentiation, heralds the early stages of osteoblast maturation and subsequent bone formation [17]. An increase in this gene's expression signifies that the composites not only support cellular proliferation but also enhance osteogenic differentiation. This effect was observed to be on par with that of the non-antibiotic loaded WH particles, indicating that the presence of ampicillin does not impinge on the intrinsic osteoinductive potential of WH. Notably, we observed an osteogenic property inherent in WH, conducive to bone reproduction. Furthermore, our study demonstrated the antimicrobial potential of WH, highlighting its suitability for tissue engineering applications with built-in antimicrobial properties.

These findings collectively suggest a synergistic relationship where WH serves as a conducive scaffold for bone growth while ampicillin embedment potentially abates bacterial colonization, without detracting from the scaffold's regenerative capabilities. Given the prevalence of post-operative infections, the antibiotic-mediated protection offered by such biomaterials could be of substantial clinical importance [18]. Our data, while promising, requires the substantiation of in vivo models and long-term studies to elucidate the therapeutic efficacy and safety of the antibiotic-loaded WH composites. Additionally, future investigations should explore the controlled release kinetics of ampicillin from the composite and its impact on prolonged antibacterial activity and bone healing dynamics. 

In the broader context of biomaterials research, the incorporation of antibiotics into carrier matrices has emerged as a promising strategy for localized drug delivery. This approach mitigates the risk of systemic toxicity while ensuring targeted therapeutic effects. For instance, Lieberman et al, have explored the use of hydroxyapatite as a carrier for antibiotics, exhibiting effective inhibition of bacterial growth in vitro [2]. Similarly, our investigation focused on loading WH with ampicillin, revealing its non-toxic nature and potential for bone regenerative purposes. While our study provides valuable insights into the biocompatibility and osteogenic properties of WH loaded with ampicillin, it is essential to acknowledge the broader implications of WH in biomedicine.

Future scope

Further exploration of this study's findings could involve investigating the kinetics of drug release from WH loaded with ampicillin to optimize its sustained therapeutic efficacy, considering factors such as pH, temperature, and material composition. Additionally, examining interactions with other cell types and tissues, particularly immune cells, would provide insights into potential immunomodulatory effects and broader applications in infection control. The scalability and manufacturing feasibility of WH-based composites also merit attention to enable their translation into clinical practice. Exploring synergistic effects with other therapeutic agents or biomaterials could enhance efficacy in tissue regeneration and infection management. Moreover, rigorous in vivo studies are necessary to validate safety and efficacy in relevant animal models, providing essential preclinical data for future clinical translation.

Limitation of the study

When contemplating the utilization of WH-loaded antibiotics, it is paramount to acknowledge the potential limitations inherent in this approach. These encompass various aspects such as biocompatibility and toxicity, drug release kinetics of both WH and ampicillin, stability of WH, antibiotic selection, mechanical properties, degradation and resorption rates, regulatory approval processes, as well as considerations regarding cost and scalability. It is imperative to approach the development and application of WH-loaded antibiotics with a comprehensive understanding of the material properties, drug release profiles, and intended therapeutic applications. Robust preclinical investigations are indispensable for addressing these limitations effectively before contemplating clinical implementation.

## Conclusions

In conclusion, the incorporation of ampicillin into WH holds promise for localized antibiotic delivery and bone regeneration. The ampicillin-loaded WH composite presents itself as a candidate with dual functionalities. The ampicillin-loaded WH is found to be nontoxic and it can be used for bone regenerative purposes. It also possesses osteogenic properties. This approach is particularly valuable in orthopedics and dentistry where implants are commonly used, and infection prevention is crucial for the success of such implants. The antibiotic-loaded WH can be used as a component in the fabrication of bone implants or drug delivery systems. It can release antibiotics gradually, providing localized treatment and reducing the risk of bacterial infections at the implant site. Our findings contribute to the growing body of literature on biomaterials research, underscoring the potential of WH-based composites in advancing therapeutic interventions in orthopedics and tissue engineering. Future studies should delve deeper into the optimization of drug release kinetics, stability, and scalability of WH-based formulations to realize their full translational potential in clinical settings.
